# Datathons and Software to Promote Reproducible Research

**DOI:** 10.2196/jmir.6365

**Published:** 2016-08-24

**Authors:** Leo Anthony Celi, Sharukh Lokhandwala, Robert Montgomery, Christopher Moses, Tristan Naumann, Tom Pollard, Daniel Spitz, Robert Stretch

**Affiliations:** ^1^ Critical Data Massachusetts Institute of Technology Cambridge, MA United States; ^2^ Beth Israel Deaconess Medical Center and Harvard Medical School Boston, MA United States; ^3^ Division of Pulmonary and Critical Care Medicine University of Washington Seattle, WA United States

**Keywords:** reproducibility of findings, big data, database, Internet, medical informatics

## Abstract

**Background:**

Datathons facilitate collaboration between clinicians, statisticians, and data scientists in order to answer important clinical questions. Previous datathons have resulted in numerous publications of interest to the critical care community and serve as a viable model for interdisciplinary collaboration.

**Objective:**

We report on an open-source software called Chatto that was created by members of our group, in the context of the second international Critical Care Datathon, held in September 2015.

**Methods:**

Datathon participants formed teams to discuss potential research questions and the methods required to address them. They were provided with the Chatto suite of tools to facilitate their teamwork. Each multidisciplinary team spent the next 2 days with clinicians working alongside data scientists to write code, extract and analyze data, and reformulate their queries in real time as needed. All projects were then presented on the last day of the datathon to a panel of judges that consisted of clinicians and scientists.

**Results:**

Use of Chatto was particularly effective in the datathon setting, enabling teams to reduce the time spent configuring their research environments to just a few minutes—a process that would normally take hours to days. Chatto continued to serve as a useful research tool after the conclusion of the datathon.

**Conclusions:**

This suite of tools fulfills two purposes: (1) facilitation of interdisciplinary teamwork through archiving and version control of datasets, analytical code, and team discussions, and (2) advancement of research reproducibility by functioning postpublication as an online environment in which independent investigators can rerun or modify analyses with relative ease. With the introduction of Chatto, we hope to solve a variety of challenges presented by collaborative data mining projects while improving research reproducibility.

## Introduction

A growing body of evidence suggests that high-quality data are lacking to guide clinician decision making. A systematic review of the joint American College of Cardiology and American Heart Association clinical practice guidelines revealed that only 314 of the 2711 recommendations were based on high-quality evidence [1]. Most of what clinicians do in practice has not been evaluated and is not covered by existing guidelines. Furthermore, it is unlikely that there will be prospective randomized controlled trials, the gold standard of evidence-based medicine, to address all the information gaps in clinical practice.

Furthermore, the reliability of published research has been called into question. One study found that among 49 highly cited articles, 16% were subsequently contradicted, and another 16% were found to have significantly smaller effect sizes in subsequent studies [2]. In order to address some of these issues, a greater emphasis has been placed on sharing data, as well as fostering a more open peer review process.

The near ubiquitous implementation of electronic health records has allowed investigators to address treatment and diagnostic dilemmas where evidence-based guidelines are lacking; however, clinicians are often lacking the increasing expertise in data science required to acquire and synthesize such dat. In order to tackle this new frontier of medical research, it is paramount that clinicians and data scientists work together to harness the power of electronic health records.

In previous publications, we described the “datathon” model, which brings together the requisite experts from different scientific disciplines in a venue that supports collaboration, group learning, error checking, and methodological review during the initial design and subsequent phases of research [3,4].

In September 2015, the second international Critical Care Datathon was held simultaneously at the Massachusetts Institute of Technology (MIT) in Boston, USA, and in London, UK and Paris, France. The event coincided with the launch of Medical Information Mart for Intensive Care (MIMIC-III), the successor to Multiparameter Intelligent Monitoring in Intensive Care (MIMIC-II), and an open-access database of patients admitted to an intensive care unit (ICU) at Beth Israel Deaconess Medical Center in Boston, MA, USA [5]. MIMIC-III spans the period from 2002 through 2012 and contains data on over 60,000 ICU admissions. Previous datathons have resulted in numerous publications of interest to the critical care communities. Using a diverse range of methodologies, groups have investigated the association between elevated central venous pressure and acute kidney injury [6], proton pump inhibition and cardiac arrhythmias [7], hyperdynamic left ventricular ejection fraction and mortality [8], diuretic use and obesity [9], and hypermagnesemia and blood pressure [10]. Pereira et al used fuzzy modeling to predict severely depressed left ventricular ejection fraction following ICU admission [11].

All participants at the 2015 datathon were encouraged to use open-source software called Chatto [12] during the event. This suite of tools was created by members of our group to fulfill two purposes: (1) facilitation of interdisciplinary teamwork through improved communication, as well as archiving and version control of datasets, analytical code, and team discussions, and (2) advancement of research reproducibility by functioning postpublication as an online environment in which independent investigators can rerun or modify analyses with relative ease. Chatto allows individual data scientists and teams to rapidly begin to explore, visualize, extract, and analyze data. Perhaps its most important role is in its simplification of the data analysis pipeline and subsequent capacity to improve reproducibility without requiring additional effort outside of the usual research workflow.

**Figure 1:**
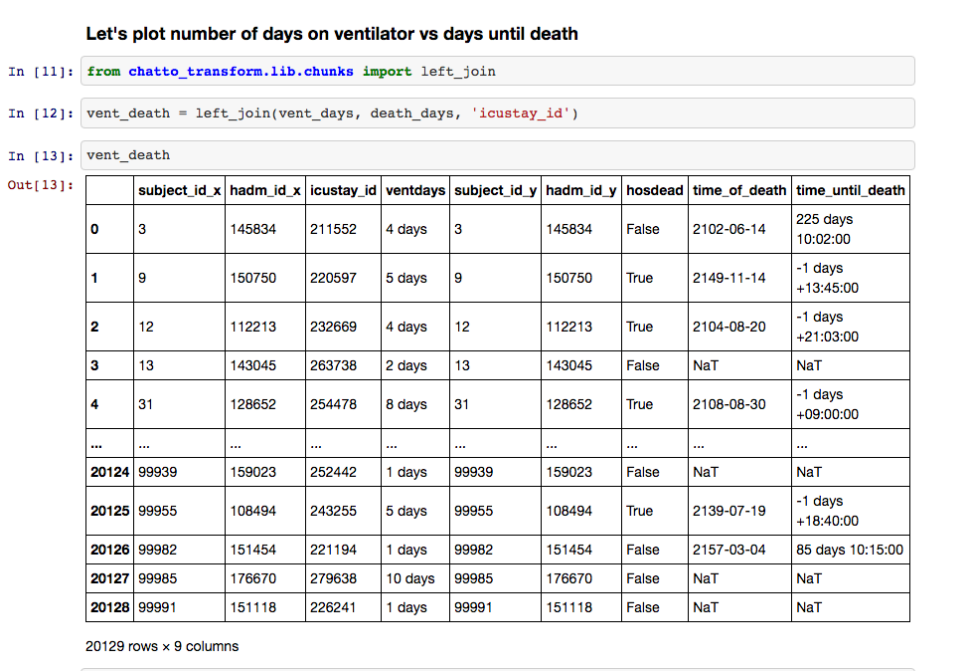
Sample workbook from Jupyter notebooks, which provide an interface for documentation of the research.

**Figure 2:**
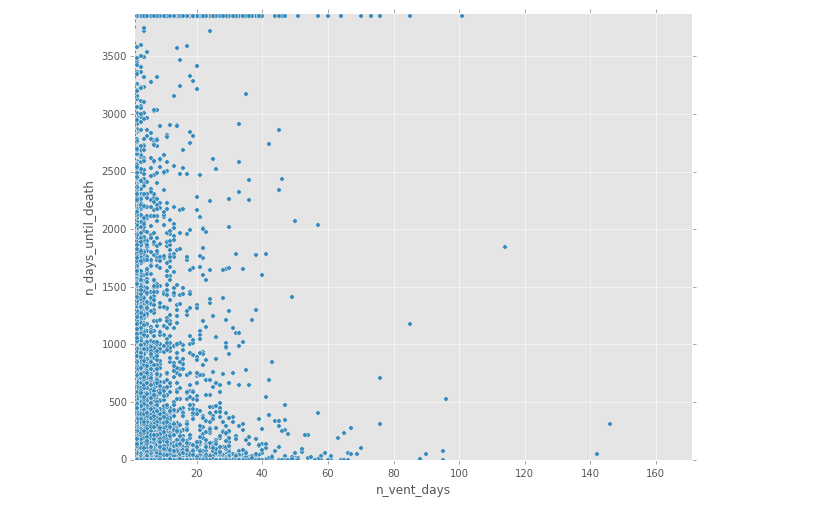
Generation of figures within Jupyter notebooks: sample scatterplot.

**Figure 3:**
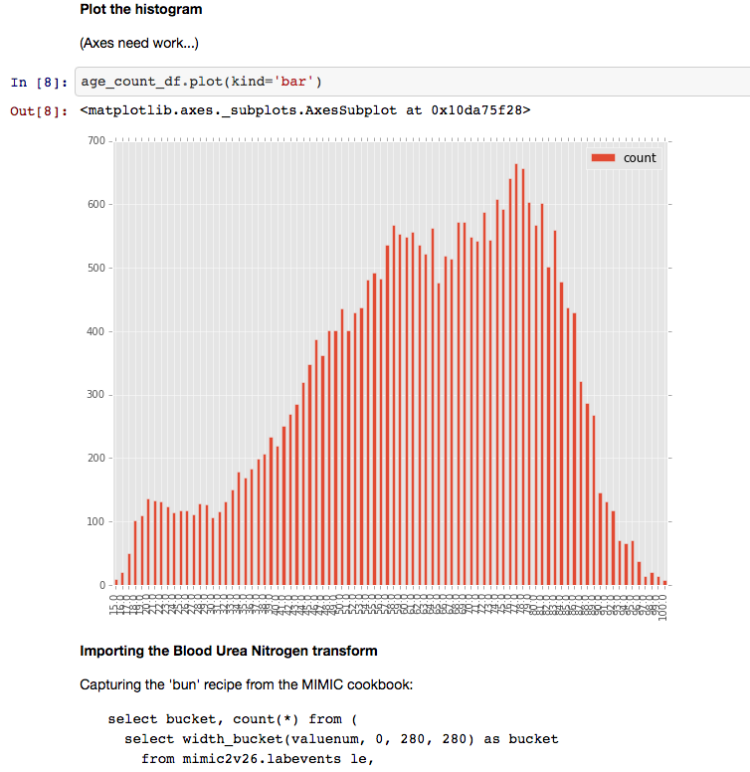
Generation of graphs within Jupyter notebooks: sample histogram.

Chatto is composed of five key components: a project website, integration with a group chat service called Slack [13], integration with GitHub [14] for source code control, a Jupyter [15,16] notebook for interactive code development, and an open-source library [12] for connecting to data sources and transforming data (Figure 1, Figure 2, Figure 3). Figure 1 shows a sample workbook. Figure 2 and Figure 3 demonstrate a scatterplot and a histogram, respectively, which were made using the provided data transformations.

## Methods

Similar to prior datathons, the event began with introductory presentations outlining the program and sharing lessons learned from prior datathons. Afterward, participants pitched various clinical questions that could be addressed using the MIMIC-III database. Teams formed organically as clinicians and data scientists discussed potential research questions and the methods required to address them. Each multidisciplinary team spent the next 2 days with clinicians working alongside data scientists to write code, extract and analyze data, and reformulate their queries in real time as needed. All projects were then presented on the last day of the datathon to a panel of judges that consisted of clinicians and scientists.

## Results

Event participants were exceptionally diverse. Of the 48 attendees at MIT, 23 (48%) were clinicians, of whom 18 (78%) were fellows, residents, or medical students. Of the 25 data scientists, 5 were postdoctoral associates (20%) and another 5 were graduate students (20%). Before the event, 32 participants (67%) had never used MIMIC and only 10 (21%) had used SQL queries frequently in their research work. Of the 8 teams, 6 subsequently submitted, and had accepted, abstracts to the American Thoracic Society international conference.

At the start of the event, all datathon participants were invited to visit a project website called Chatto Hub to register their team’s project in a listing alongside other datathon projects. Chatto Hub users were authenticated using GitHub user credentials. Integration with the GitHub application program interface (API) enabled a public code repository to be automatically created for each team upon project registration, based on a clone of a common parent repository that included code examples and documentation. Using the Slack API, a group chat “channel” was also automatically created for each team upon project registration, providing a means for each team to communicate and share files. Lastly, private Jupyter notebooks—browser-based interactive coding environments for data analysis and documentation—were automatically created for each user on each registered project.

The Jupyter notebooks were preconfigured to connect to a cloud-based instance of the MIMIC-III database hosted by Amazon Web Services (Amazon.com, Seattle, WA, USA). Each notebook also included MIMIC-specific data transformations—modular, reusable pieces of code—as well as the tools to create new transformations that could later be shared with the broader research community through GitHub. The result was a project listing on the Chatto Hub website that included the project title, short description, and links to both the project’s Slack channel and GitHub repository.

Use of Chatto was particularly effective in the datathon setting, enabling teams to reduce the time spent configuring their research environments to just a few minutes—a process that would normally take hours to days. Chatto continued to serve as a useful research tool after the conclusion of the datathon. The Slack implementation allowed teams to continue to collaborate in a manner that was automatically documented and accessible to all team members. Furthermore, simplification of the data analysis pipeline through the use of Jupyter notebooks means that code published alongside each study is more easily interpretable. Independent researchers will also be able to rerun or modify the original analytical code with minimal effort.

## Discussion

The issue of reproducibility among scientific publications has generated substantial concern in both the research and lay communities. Increased use of large, publicly accessible datasets in medical research has, to some extent, facilitated recognition of this problem by enabling independent investigators to reanalyze data at minimal cost. Nevertheless, the majority of published studies are not externally validated in this manner due to the persistence of significant technical and cultural barriers. These impediments include time-intensive configuration, inadequate documentation, code rot, “dependency hell,” and a research environment that insufficiently rewards efforts to reproduce the work of other investigators.

Breaking down the technical barriers that stand in the way of research reproducibility is a ripe goal. For research involving large datasets, we have developed an online open-source tool that serves as a development environment leading up to the time of publication, while functioning postpublication as a playground for independent investigators to rerun the analysis, en bloc or piecemeal, with or without modification. As a proof-of-concept, this tool was used successfully in tandem with the MIMIC-III database during the most recent MIT Critical Care Datathon (September 2015), although connectors to any large research database can be easily generated.

As tools such as the one we have described continue to evolve, the responsibility of ensuring that the analytical code related to a given study is accessible, interpretable, and “runnable” with minimal effort should increasingly fall on the original investigators. The results of studies where authors have made inadequate efforts to enable and encourage others to examine, rerun, and modify their analysis should be viewed with prejudice, and their suitability for publication should be questioned.

### Limitations

Despite the many benefits of integrating the services outlined above into a single software package, not all issues related to collaborative data mining were solved. Teams developed different methods for sharing the results of their analyses, including exporting intermediate results to comma separated value (CSV) files for distribution through the team’s Slack channel. This highlighted that teams often divided their labor based on expertise, requiring each team member’s results to be shared and then combined by one individual for more advanced analysis. In the future, enabling multiple team members to simultaneously edit a shared Jupyter notebook might help to resolve this problem. Jupyter notebooks were also not automatically source controlled. In the future, GitHub integration for hosted notebooks would dramatically simplify the research workflow and sharing of analytical code between team members. Lastly, although documentation existed for both MIMIC-III and each individual service integrated with Chatto, the lack of a single centralized location for this documentation presented a problem for some participants. This issue was mitigated during the event by datathon staff actively helping participants to troubleshoot technical problems, but this should be addressed through centralized, simplified documentation in future datathons.

### Conclusion

The product of scientific research is not a number with an accompanying *P* value, but rather a thorough demonstration of the method through which a conclusion was reached from a given set of data with the ultimate goal of improving patient outcomes and quality of care. As analytical methods becoming increasingly sophisticated and datasets grow in size and complexity, we must not lose sight of the importance of enabling independent researchers to validate the findings of their peers without requiring them to reinvent the wheel.

## Abbreviations

API: application program interface
CSV: comma separated value
ICU: intensive care unit
MIMIC-II: Multiparameter Intelligent Monitoring in Intensive Care
MIMIC-III: Medical Information Mart for Intensive Care
MIT: Massachusetts Institute of Technology
